# Modulation of Sleep Homeostasis by Corticotropin Releasing Hormone in REM Sleep-Deprived Rats

**DOI:** 10.1155/2010/326151

**Published:** 2010-06-13

**Authors:** Ricardo Borges Machado, Sergio Tufik, Deborah Suchecki

**Affiliations:** Departamento de Psicobiologia, Universidade Federal de São Paulo, 04024-002 São Paulo, Brazil

## Abstract

Studies have shown that sleep recovery following different protocols of forced waking varies according to the level of stress inherent to each method. Sleep deprivation activates the hypothalamic-pituitary-adrenal axis and increased corticotropin-releasing hormone (CRH) impairs sleep. The purpose of the present study was to evaluate how manipulations of the CRH system during the sleep deprivation period interferes with subsequent sleep rebound. Throughout 96 hours of sleep deprivation, separate groups of rats were treated i.c.v. with vehicle, CRH or with alphahelical CRH_9−41_, a CRH receptor blocker, twice/day, at 07:00 h and 19:00 h. Both treatments impaired sleep homeostasis, especially in regards to length of rapid eye movement sleep (REM) and theta/delta ratio and induced a later decrease in NREM and REM sleep and increased waking bouts. These changes suggest that activation of the CRH system impact negatively on the homeostatic sleep response to prolonged forced waking. These results indicate that indeed, activation of the HPA axis—at least at the hypothalamic level—is capable to reduce the sleep rebound induced by sleep deprivation.

## 1. Introduction

One of the most interesting sleep phenomena is its homeostatic regulation, which can be manifest by the rebound in sleep that ensues after total or partial sleep deprivation, for example, increased time spent in sleep during the recovery period [1]. This phenomenon, known as sleep rebound, is also observed after deprivation of selected sleep stages, when recovery of the suppressed stage is observed [2]. However, sleep deprivation is considered a form of stress both in humans [3] and rats (for review, see [4]), although there is not complete agreement on the matter [5]. Animal models of sleep deprivation indicate that not only the loss of sleep per se, but also the method employed to induce sleep deprivation generates stress, resulting in increased activity of the hypothalamic-pituitary-adrenal (HPA) axis, with elevated corticosterone (CORT) and adrenocorticotropic (ACTH) plasma levels and adrenal hypertrophy [6–9]. Additional data demonstrate that sleep deprivation induces increased immunoreactivity [10] and expression of hypothalamic CRH [11].

 Interestingly stressors can induce specific changes in sleep patterns, including increased REM sleep after immobilization stress [12], increased slow wave sleep after social defeat stress [13], and decreased REM sleep after footshock [14–16]. When associated with sleep deprivation, however, immobilization stress inhibits the homeostatic REM sleep rebound [17], whereas intermittent chronic foostshock exacerbates the expression of REM sleep during recovery, in an apparently prolactin- and CORT-dependent effect [18]. Curiously, both exogenous corticosterone administration and dexamethasone treatment inhibit sleep in unstressed rats [19, 20] or after immobilization stress [21], indicating that other mediators participate in this phenomenon. Corticotropin-releasing hormone (CRH), the primary orchestrator of the endocrine stress response, is synthesized in the paraventricular nucleus of the hypothalamus [22] and is a major regulator of waking in rats [23–25]. It increases neuronal excitability and convulsions [26], and stimulates the locus coeruleus noradrenergic neurons [27]. CRH receptors are densely distributed in basal prosencephalic areas, thalamus, hypothalamus, mesencephalus, brainstem, and pons [28], areas which are involved in cerebral activation and waking maintenance [29].

The pioneering study by Ehlers and coworkers [25] on the effects of CRH on sleep in rats demonstrates that low doses of this peptide reduce slow wave sleep (NREM) and low frequency activity. However, in high doses, CRH exhibits an opposite effect and reduces fast frequency activity (32–64 Hz). In human beings, however, peripheral administration of CRH does not significantly alter REM sleep [30]. Moreover, CRH modulates the homeostatic rebound induced by sleep deprivation, by increasing REM sleep rebound when administered immediately after the sleep deprivation procedure [31]. In contrast, *α*-helical-CRH_9−41_ (*α*hCRH), a CRH receptor blocker, prevents immobilization stress-induced sleep rebound and does not influence sleep in stress-free conditions [32].

In an attempt to determine the role of hormones of the HPA axis, we recently demonstrated that 96 hours of REM sleep deprivation together with repeated administrations of metyrapone, a corticosterone synthesis inhibitor, impaired sleep deprivation-induced NREM compensation [33]. We then hypothesized that increased production of CRH elicited by removal of the CORT negative feedback signal at the hypothalamic level [34] could be, at least in part, responsible for this effect. If this hypothesis were correct, then i.c.v. administration of CRH in sleep-deprived rats should produce similar results to those obtained with metyrapone, and *α*-helical-CRH_9−41_ and should increase sleep rebound in these animals.

## 2. Methods

### 2.1. Subjects

Male adult Wistar rats (350–450 g) from the animal facility of the Department of Psychobiology—UNIFESP—were used (eight to ten animals per group) and prior approval from the Ethics Research Committee of Universidade Federal de São Paulo was obtained in accordance with international guidelines for care in animal research (CEP 0125/04). Constant 12 hours light-dark cycle (fluorescent white lamps-lights on at 7:00 h) and 20–22°C temperature were maintained in all experimental rooms throughout the experimental protocol. Rats were allowed free access to food and water.

### 2.2. Electrophysiological Procedures

Under ketamine-xylazine anaesthesia (9.0–10.5 mg/kg, i.p.), rats were fitted with electrodes to monitor the sleep-wake cycle: two bipolar electrodes placed ipsilaterally with stainless-steel micro-screws (0.9 mm in diameter) were used for EEG monitoring: one pair on the right lateral parietoparietal (for minimum theta activity EEG) and the other on the left medial frontoparietal (for maximum theta activity EEG) areas [35, 36]. One pair of insulated nickel-chromium flexible fine wire electrodes was implanted in the dorsal neck muscle for EMG recording. For intracerebroventricular—i.c.v. injections, a 22-gauge stainless steel guide cannula (constructed from hypodermic needle, Becton Dickinson, Brazil, cut at 10 mm in length) was inserted 1.0 mm posterior to bregma, 1.4 mm lateral to midline, and 3.4 mm ventral to dura membrane, within the EEG electrodes. The guide cannula was covered with an easily removable lid adopted from a fine stainless steel wire, which was inserted tightly onto the guide cannula hole. After the surgical procedure, antibiotics (Pentabiótico Fort-Dodge, Brazil) and sodium diclofenac were administered and the animal was allowed to recover from surgery for 15 days. Three days before the beginning of experiments, lateral ventricular cannula placement was verified by assessing the drinking response elicited to up to 5 nmol angiotensin II administration. After the multiple injections schedules, the ventricular cannula placement was confirmed postmortem by injection 3 *μ*L of methylene blue followed by microscope visualization. Animals were habituated to the cables and to the recording environment for 3 days before baseline recording. Baseline sleep was recorded on two consecutive days (2 × 24 hours) and the parameters are represented by the average of these two days. After the baseline recording, in the period that preceded REM sleep deprivation (REMSD), animals were adapted to the sleep deprivation chambers for 30 minutes per day for three consecutive days.

Electrophysiological signals were recorded on a digital polygraph (Neurofax QP 223 A Nihon Kohden Co., Tokyo, Japan). After conventional amplification, the EEG signals were conditioned through analog filters, using cut off frequencies of 1.0 Hz and 35.0 Hz and were then sampled at 200 Hz using a 16 bit A/D converter. Recordings were displayed on 10 s epochs and submitted off-line to visual scoring routine, as described previously [37]. In summary the stages of wake-sleep were defined as follows: (a) waking (low voltage and high frequency EEG, whereas EMG displays high voltage during active waking or low during voltage quiet waking); (b) NREM sleep (EEG high voltage within slow waves and spindles, also classified, separately, in low—between 2.0 and 3.0 *μ*V, and high amplitudes—from 3.0 *μ*V on, and low EMG amplitude); and (3) REM sleep (low EEG voltage with prominent theta rhythm on medial EEG deviation accompanied of the very low EMG activity). Each 10 s epoch was characterized by the predominant wave pattern present in more than half of the epoch. In some periods, interference or noise made it impossible to characterize the behavioral state and a critical evaluation of the previous and subsequent periods was made. The percentage of 10 s periods excluded from sleep scoring was below 9%.

The parameters used for sleep analysis were the following: total sleep time, total NREM time (considering low and high amplitude fractions) REM time and bouts, and total wake time—active and quiet periods—and episodes of waking (2.0 minutes). Fast Fourier Transform (Hanning window) was computed on 256 points for each 10 s epochs (corresponding to each vigilance state) with a resolution of 0.78 Hz (null value was attributed to the remaining time). Nonoverlapping bands were set giving 0.5 Hz bins from 1.0 to 5.0 Hz, and 1.0 Hz bins from 5.1 Hz to 25.0 Hz; those above 25.0 Hz were discarded from the analysis. EEG epochs containing noise or artifacts (those that did not allow doubtful behavioral state classification) were excluded from the analysis by visual inspection and/or spectral tools (e.g., if average power exceeded 2000 *μ*V^2^ over a 1.0–25.0 Hz frequency range). Slow wave activity was calculated as mean power density on 1.0–4.0 Hz band (delta) and the theta-delta ratio, dividing the power density of the fast theta (6.0–9.0 Hz) band by the mean power density on the delta band. Although two ipsilateral bipolar electrodes were used for sleep scoring, only the lateral parietoparietal deviation was used for spectral data analysis, except for the theta-delta ratio, when the two deviations were employed (the lateral to delta and the medial frontoparietal to theta activity measures).

### 2.3. REM Sleep Deprivation (REMSD) Procedure and Drug Administration

Sleep deprivation was accomplished by the single platform method, in which each animal was placed onto a narrow cylindrical platform, 6.5 cm in diameter, surrounded by water to about 1 cm below the platform surface. This method is well known to selectively suppress REM sleep; however, it also produces partial NREM deprivation, with 37–50% reduction from baseline levels [38, 39]. 

CRH (Sigma, USA): 3 *μ*g/animal [22, 40, 41] or *α*hCRH (Sigma, USA): 20 *μ*g/animal [42–44] were diluted in artificial cerebrospinal fluid—ACSF (NaCl 127 mM, KCl 2.5 mM, MgCl_2_ 0.9 mM, Na_2_HPO_4_ 1.2 mM, CaCl_2_ 1.3 mM, NaHCO_3_ 21 mM, C_6_H_12_O_6_ 3.4 mM, and pH 7.3, sterile- and pyrogen- free) and administered twice/day, at 7:00 h and 19:00 h, in addition to a single injection at the end of the REM deprivation period, making a total of nine administrations. Control animals were treated with ACSF under the same scheme. Final volume of each i.c.v. injection was 3-4 *μ*L, delivered at a flow rate of 1.5 *μ*L/minute with an injection cannula, made from another 30-gauge dentist needle (Becton Dickinson, Brazil, cut at 25 mm in length, but inserted into guide cannula up to 10.5 mm limit) connected to a polypropylene tubing (PE 10, Becton Dickinson, USA) which, in turn, was linked on another terminal end to a 10 *μ*L microsyringe (Hamilton, USA), placed onto a automatic microinfusion pump (Insight, Brazil). Prophylactic aseptic techniques were strictly employed during all administrations and less than 20% of animals were not used during the chronic experiments because of signs of infection (e.g., fever, weight loss, apathy, wet fur, and poor physical appearance). After four days under this protocol, rats were returned to sleep freely in their individual home cages (recovery period). Over the subsequent three days, the rats were continuously monitored.

### 2.4. Plasma Hormone Determination

Trunk blood was obtained by decapitation, approximately 2 hours after the last administration from matched groups, run simultaneously with the sleep study. During this period, the REMSD animals were not allowed to sleep (they were put back into the deprivation chambers). Blood was collected in chilled K_2_EDTA (0.46 mM, e.g., 7.5% solution at a volume of 0.1 mL diluted in 5 mL of blood)-containing vials, centrifuged at 2300 rpm at 4°C for 20 minutes, and plasma was collected and frozen at −20°C for further analysis. Plasma ACTH was determined by sequential immunometric assay (DPC Immulite, Los Angeles, CA) and the sensitivity of the method is 9 pg/mL, and intra- and interassay variations are 9.4% and 9.6%, respectively. Corticosterone levels were assayed by specific radioimmunoassay (INC Biomedicals, Costa Mesa, CA). The sensitivity of the assay is 1.25 ng/mL and the intra- and inter-assay variations are, respectively 6.5% and 7.1%, as informed by the manufacturer. All samples were assayed in duplicate.

### 2.5. Statistics

Hormonal data were analyzed by a two-way ANOVA, with main factor Group (CTL—control home cage and REM sleep deprivation—REMSD) and Treatment (ACSF, CRH, and *α*hCRH). Sleep parameters were analyzed by a two-way ANOVA for repeated measures, with main factors Treatment (ACSF, CRH, and *α*hCRH) and Day (repeated measure: Baseline and Recovery days 1 [R1], 2 [R2], and 3 [R3]). The spectra power density was analyzed by Student's *t* tests for independent samples, every 12 hours period, for each behavioral state separately. The theta-delta ratio was analyzed by covariance analyses (ANCOVA) where the baseline index was the predictive factor and treatments, the independent variable. All EEG data were analyzed during the light and dark phases, separately. Posthoc analysis was performed by the Newman-Keuls test. The level of significance was set at *P* ≤ .05.

## 3. Results

### 3.1. HPA Axis Hormones (Figure 1)

**ACTH**

There was a significant interaction between Group and Treatment (*F*_2,54_ = 0.67, *P* ≤ .0005), in which REMSD + ACSF animals showed higher ACTH levels than their CTL counterparts (*P* ≤ .05), whereas REMSD+ *α*hCRH animals exhibited lower levels than CTL+ *α*hCRH ones (*P* ≤ .05). In addition, in CTL animals, both CRH and *α*hCRH resulted in higher ACTH levels than ACSF administration (*P* ≤ .0005).

**Corticosterone**

Again, a significant interaction between Group and Treatment (*F*_2,53_ = 19.52, *P* ≤ .00005) was found. Newman-Keuls analysis of this interaction showed that all groups exhibited higher corticosterone plasma levels than CTL + ACSF animals (CTL+CRH: 853.3%, *P* ≤ .0005; CTL + *α*hCRH: 230.5%, *P* ≤ .05; REMSD + ACSF: 115.4%, *P* ≤ .05; REMSD + CRH: 575.6%, *P* ≤ .0005 and REMSD + *α*hCRH: 747.6%, *P* ≤ .0005). However, CRH treatment in REMSD rats resulted in lower CORT levels than in CTL rats (−29.1%, *P* ≤ .01), although they were still higher than REMSD+ACSF animals (213.6%, *P* ≤ .0005). On the other hand, *α*hCRH treatment led to higher CORT levels in REMSD than in CTL (156.4%, *P* ≤ .0005) and REMSD + ACSF rats (293.5%, *P* ≤ .0005). Finally, CORT concentrations were lower in CTL + *α*hCRH than in CTL+CRH (−65.33%, *P* ≤ .0005).

### 3.2. Sleep Parameters

#### 3.2.1. Total Sleep Time (Figure 2)

**Light Phase**

A main effect of Day (*F*_3,63_ = 2.52, *P* ≤ .00001) was found. Reduced sleep time was observed on the 2nd(10.2%, *P* ≤ .05) and 3rd recovery days when compared to baseline (22.9%, *P* ≤ .0005).

**Dark Phase**

Again main effect of Day was observed (*F*_3,63_ = 13.89, *P* ≤ .00001). Animals showed 24.4% increased sleep time on the first recovery day (*P* ≤ .001).

#### 3.2.2. Total NREM Time (Figure 2)

**Light Phase**

A main effect of Day was detected (*F*_3,63_ = 6.84, *P* ≤ .0005). Total NREM was reduced (20.3%) on the 3rd recovery day, relative to baseline.

**Dark Phase**

An effect of Day emerged (*F*_3,63_ = 9.05, *P* ≤ .00005) and the rats showed more NREM (16.8%, *P* ≤ .01) than baseline.

#### 3.2.3. REM Sleep Time (Figure 3)

**Light Phase**

A main effect of Day was found (*F*_3,63_ = 22.70, *P* ≤ .00001). Animals showed an increase on this phase (53.3%, *P* ≤ .0005) during the first recovery day, whereas a reduction of REM was also observed during the last recovery day (32.8%, *P* ≤ .02).

**Dark Phase**

Again, a main effect of Day was found (*F*_3,63_ = 15.32, *P* ≤ .00001) and the animals showed an increase of 71.2% (*P* ≤ .0005) during first recovery day, when compared to baseline amounts.

#### 3.2.4. REM Bouts (Figure 3)

**Light Phase**

A main effect of Day was found (*F*_3,63_ = 11.29, *P* ≤ .00001) and a decrease of REM bouts was detected on the 3rd recovery day (39.3%, *P* ≤ .0005).

**Dark Phase**

Again, a main effect of Day was detected (*F*_3,63_ = 7.83, *P* ≤ .0005). During the first recovery day, animals displayed more bouts than baseline sleep (*P* ≤ .01).

#### 3.2.5. Mean Length of REM Episodes (Figure 3)

**Light Phase**

A two-way interaction between Day and Treatment was found (*F*_6,63_ = 3.59, *P* ≤ .005). Posthoc analysis showed that REM episodes were longer after ACSF administration than at baseline (124.2%, *P* ≤ .0005). Administration of CRH (45.5%, *P* ≤ .005) and *α*hCRH (40.0%, *P* ≤ .005) shortened the length of REM episodes during the first recovery day compared to ACSF-treated rats.

**Dark Phase**

There was a Day effect (*F*_3,57_ = 4.10, *P* ≤ .02), in which the animals showed longer REM episodes on the first recovery night (28.7%, *P* ≤ .05).

#### 3.2.6. Total Wake Time (Figure 4)

**Light Phase**

A two-way interaction between Day and Treatment was detected (*F*_6,63_ = 3.53, *P* ≤ .005) and post-hoc tests revealed that CRH-treated animals spent more time awake during the last recovery day than in baseline (41.8%, *P* ≤ .01).

**Dark Phase**

A two-way interaction was found between Day and Treatment (*F*_6,63_ = 2.94, *P* ≤ .02) and both CRH- and *α*hCRH-treated rats showed less total wake during the first recovery day than on baseline (19.1%, *P* ≤ .05 and 21.4%, *P* ≤ .05, resp.).

#### 3.2.7. Number of Awakenings (Figure 4)

**Light Phase**

A two-way interaction was found (*F*_6,63_ = 2.61, *P* ≤ .05). Post-hoc tests showed that CRH-treated rats displayed more events of awakenings during the last recovery day relative to baseline (62.6%, *P* ≤ .05).

**Dark Phase**

No changes were found.

### 3.3. Spectral Data

#### 3.3.1. Spectral Power during NREM (Figure 5)

**Light Phase**

During the third recovery day, CRH-treated animals showed reductions in the 19.01–20.0 Hz (19.2%, *P* ≤ .05) and 20.1–21.0 Hz bands (18.4%, *P* ≤ .05) when compared to ACSF-treated rats. Moreover, increased power above ACSF animals was observed in the 1.6–2.0 Hz band (53.3%, *P* ≤ .02) in CRH- and in the 1.0–1.5 Hz band (34.8%, *P* ≤ .05) in *α*hCRH-treated groups.

**Dark Phase**

On the second recovery day, *α*hCRH led to increased power in the 1.0–3.0 Hz bands (average of 4 bins of 0.5 Hz each = 53.7%, *P* ≤ .05), when compared to ACSF group and increased all bands above 6.0 Hz when compared to CRH-treated rats (average of 19 bins of 1.0 Hz each = 28.5%, *P* ≤ .05). Finally, during the last recovery day, *α*hCRH produced an increase in all bands above 7.0 Hz when compared to CRH-treated animals (average of 18 bins of 1.0 Hz each = 27.1%, *P* ≤ .05).

#### 3.3.2. Theta-Delta Ratio (Figure 6)

**Light Phase**

A main effect of Treatment was detected for active wake (AW) (*F*_2,20_ = 4.45, *P* ≤ .05) and post-hoc analysis showed that CRH animals showed reductions of *θ*/*δ* relative to ACSF (12.3%, *P* ≤ .05) and *α*hCHR rats (17.6%, *P* ≤ .01). During Quiet Wake (QW), effect of Treatment was again revealed (*F*_2,19_ = 3.94, *P* ≤ .05), and CRH-displayed smaller *θ*/*δ* ratio than *α*hCHR-treated animals (16.8%, *P* ≤ .01). No effects were found in Low-Amplitude NREM (L-NREM) or in High-Amplitude NREM (H-NREM) during the recovery period. During REM sleep, a significant effect of Treatment (F_2,20_ = 7.14, *P* ≤ .005) and an interaction between Day and Treatment (F_4,40_ = 2.90, *P* ≤ .05) were found. In regards to the Treatment factor, CRH-treated animals showed a smaller *θ*/*δ* ratio than ACSF- (15.6%, *P* ≤ .01) and *α*hCHR-treated animals (22.1%, *P* ≤ .005). No post-hoc differences were detected for the interaction.

**Dark Phase**

During AW a main effect of Day was observed (*F*_2,40_ = 12.61, *P* ≤ .00005); however, the post-hoc analysis did not revealed any differences among the days. For QW, main effects of Treatment (*F*_2,20_ = 3.59, *P* ≤ .05) and Day (*F*_2,40_ = 10.27, *P* ≤ .0005) were found. The Newman-Keuls test showed that CRH treatment reduced *θ*/*δ* ratio when compared to ACSF (9.7%, *P* ≤ .05) and *α*hCHR (15.0%, *P* ≤ .005) treatments. In regards to the effect of Day, there was a reduction of *θ*/*δ* on the 3rd compared to the 1st recovery day (6.1%, *P* ≤ .05). No effects were detected in L-NREM, whereas a main effect of Day was detected in H-NREM (*F*_2.40_ = 3.35, *P* ≤ .05) and the post-hoc analysis showed that the *θ*/*δ* ratio was reduced on the 2nd and 3rd recovery days (9.7%, *P* ≤ .0001 and 13.7%, *P* ≤ .0001, resp.). During REM sleep, a main effect of Treatment was detected (*F*_2,18_ = 4.00, *P* ≤ .05) and CRH treatment reduced the *θ*/*δ* ratio compared to ACSF (12.4%, *P* ≤ .05) and to *α*hCHR (17.1%, *P* ≤ .01).

## 4. Discussion

The main results of the present study can be summarized as follows: (1) both CRH and *α*hCRH increased ACTH and CORT secretions, although these were lower in REMSD than in control rats; (2) both peptides impaired sleep in later periods of the recovery sleep, but did not interfere with the immediate sleep rebound, except for a reduction in the length of REM sleep episodes; (3) rats treated with *α*hCRH exhibited more high frequency bands in NREM than rats treated with CRH during the last dark phase of the recovery period; and (4) CRH-treated rats exhibited lower theta/delta ratio, indicating an impairment of the homeostatic sleep rebound. 

As expected, REMSD resulted in increased secretion of ACTH and CORT levels, relative to control, nondeprived rats. Repeated administration of CRH, during REMSD, however, led to opposite effects, for example, levels in REMSD were lower than those of control rats. This result can be explained by the well-known stimulating effect that REMSD exerts on the CRH-producing neurons, with increased mRNA [11] and immunoreactivity of CRH [10] in the PVN, which could lead to lower density of CRH receptors. In fact, a previous study showed that REMSD results in lower CRH receptor density in the pituitary and striatum [45]. Despite that, no major effects in the sleep macrostructure were observed during rebound, except for a reduction of the length of REMS episodes.

 We found that *α*hCRH reduced ACTH, but increased CORT release by almost 3-fold in REMSD rats compared to CSF-treated animals. Alpha-helical CRH-induced attenuation of the ACTH response to REMSD resembled those of a previous study with restraint stress [46]. Some studies indicate that in order to achieve an effective blockade of the behavioral stress response, *α*hCRH dose must be higher than 25 *μ*g (in the present study the dose was 20 *μ*g/animal) [42, 47, 48], even though ACTH and CORT secretions may still not be completely suppressed [42]. Some studies even indicate that *α*hCRH, in doses higher than 25 *μ*g, acts as an agonist of the type 1 CRH receptor (CRH-R1), leading to behavioral and hormonal responses similar to those elicited by CRH [49–52]. *α*hCRH antagonist action is evident in stressful, but not under basal conditions [53–55], possibly due to the fact that it binds more efficiently to CRH-R2 receptors [56], whereas CRH-R1 is the predominant type in the pituitary [57–59]. Interestingly, the distribution of CRH receptors in sleep-related areas indicates that CRH-R1 is densely located in the laterodorsal tegmental nucleus and CRH-R2, in the dorsal raphe, without any overlapping [59], suggesting that both receptors types may be involved in sleep regulation.

Increased NREMS is seen with higher doses of *α*hCRH (i.c.v., 25 *μ*g/rat) within two hours of drug administration [43], whereas a higher dose (i.c.v., 100 *μ*g/rat) prevents immobilization stress- and sleep deprivation-induced sleep rebound [32, 60]. It is possible that the dose of *α*hCRH used in the present study, also infused i.c.v., was not high enough to produce the same sleep changes as reported by Gonzalez and Valatx's [60] paper, however, in their study, the compound was administered every two hours throughout the deprivation period, mounting to a much larger dose in a much shorter period of sleep deprivation. Moreover, this schedule of administration is likely to maintain receptors blocked throughout the entire sleep deprivation period.

The most remarkable effect of CRH and *α*hCRH treatments in sleep macrostructure was a shortening of the length of REMS episodes, compared with vehicle-infused rats, during the first light period of recovery sleep, indicating and impairment of REM sleep regulation. The influence of CRH on REM sleep appears to be bimodal. On the one hand, intra-hippocampal CRH infusion reduces theta rhythm by acting on both CRH receptors, present in hippocampal CA1 field and dentate gyrus [61]. On the other hand, overexpression of CRH leads to more spontaneous and sleep deprivation-induced REM sleep [62]. At present, it is not possible to determine how both drugs, acting predominantly through different CRH receptors, could lead to similar results in REM sleep regulation. One possibility may involve changes in serotonergic transmission, since it has been shown that the raphe nucleus projects heavily to the hippocampus and medial septum [63, 64] and that stimulation of this region suppresses theta rhythm in the EEG, regardless of the activity in the septal area [65, 66]. Lesions of the raphe may result in permanent hippocampal theta rhythm [67] and infusion of 5-HT_1A_ agonist in the dorsomedial raphe impairs hippocampal and cortical theta rhythm [68]. Considering that the predominant CRH receptor in the raphe is the low affinity CRH-R2 [61] and that activation of these receptors with high or repeated *α*hCRH administrations lead to serotonin release in this area [69], there is a possibility that the treatment used in the present study might have caused an increase in serotonergic activity, which impaired theta rhythm and, consequently, REM sleep.

Regarding sleep microstructure, during NREM sleep, *α*hCRH produced an increase in the high frequency bands during the last two dark phases, compared to CRH, suggesting opposite homeostatic and late circadian responses exerted by these peptides. Thus, *α*hCRH-treated rats appeared to exhibit shallower NREM sleep than CRH-treated rats, considering that high frequency bands are predominant during waking. However, shallower NREM sleep during the active period of rats indicates normal circadian rhythm and, therefore, a return to homeostasis. CRH also reduces low frequency (1.0–6.0 Hz) spectral potency in rats [25] and in humans, there is an increase in waking and delta sleep EEG sigma band (11.0–15.0 Hz) [70]. CRH receptors are present in several thalamic nuclei [71, 72], although they inhibits spontaneous activity of these neurons [73]. Activation of these receptors might result in inactivity of reticular cells, responsible for the generation of synchronization of low frequency waves in the cortical EEG [74] and reduction of the low frequency power spectrum [25]. CRH deleterious effects on sleep appear to be mediated by CRH-R1, since R129919, a specific CRH-R1 antagonist increases slow wave sleep in depressed patients [75]. This may explain why *α*hCRH did not affect low frequency power spectrum, since this substance blocks preferentially the type 2 CRH receptor. Moreover, CRH mRNA expression on the posterior nucleus of the thalamus is augmented during the rat resting period [76, 77]. Activation of this nucleus is related to the generation of high frequency *β* waves and suppression of *δ* and spindle activity during slow wave sleep [78, 79]. Collectively, these data could explain the increased potency of EEG high frequency bands. 

A relatively recent index is the theta/delta (*θ*/*δ*) ratio, which represents a marker of homeostatic sleep compensation and is known to be increased after sleep deprivation [80, 81], being characteristic of each sleep phase [82]. In humans, for instance, this increase takes place during the dark period, which corresponds to the resting period [83]. The reduction of *θ*/*δ* ratio in CRH-treated rats during waking and REM sleep suggests that the homeostatic compensation is flawed, because during REM sleep decreased delta and/or increased theta activity is supposed to occur.

Theta rhythm is one of the most prominent features of REM sleep in the rat [35, 84], which is generated by cell populations that flow to CA1 stratum oriens and dentage gyrus stratum molecular [85, 86]. The reduction of theta/delta rhythm in CRH-treated rats might have occurred due to a reduction of theta potency, rather than an increase in delta power. The reason for this conclusion is threefold: (1) the reduction of the index took place during waking and REM sleep, when theta predominates; (2) there were no changes during low and high NREM sleep, when delta predominates; and (3) as a general rule, low frequencies (1.0–5.0 Hz) were unchanged during NREM sleep. 

In conclusion, chronic CRH administration during REM sleep deprivation impaired the homeostatic compensation phenomenon, likely due to its excitatory action on neuronal tissue. This impairment occurred in the sleep macrostructure, with shortening of REM sleep episodes, as well as in the microstructure, in later phases of the recovery period. Because *α*hCRH acts at the CRH-R2 and may have agonistic properties when repeatedly administered in high doses, it produced paradoxical changes on hormone secretion and sleep homeostasis, being, sometimes, similar to CRH.

## Figures and Tables

**Figure 1:**
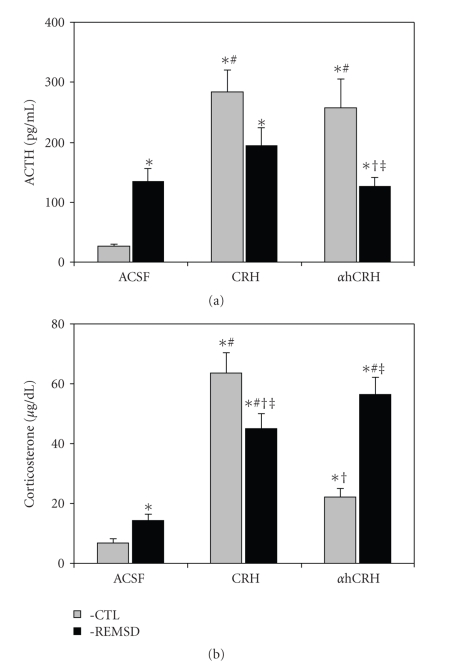
*ACTH and CORT Plasma Levels*. CTL, Control; ACSF, artificial cerebrospinal fluid; CRH, corticotrophin-releasing hormone; *α*hCRH, alpha-helical CRH_9−41_ *- different from CTL+ACSF, ^#^- different from PSD+ACSF, and ^†^- different from CTL+CRH, ^‡^- different from CTL+*α*hCRH; ANOVA, followed by Newman-Keuls test, *P* ≤ .05.

**Figure 2:**
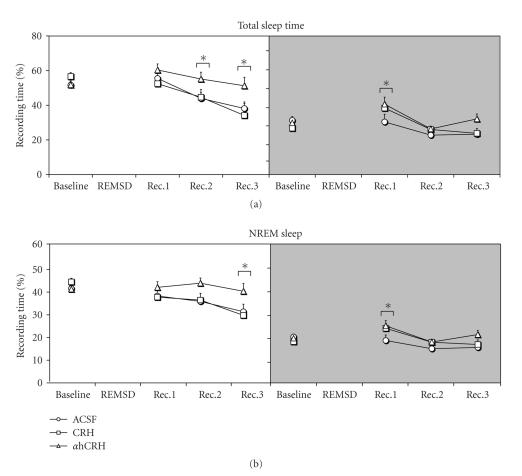
*NREM Sleep*. Data were obtained in recording periods of approximately 11 hours during the light and dark phases. ACSF, artificial cerebrospinal fluid; CRH, corticotrophin-releasing hormone; *α*hCRH, alpha helical CRH_9−41_; REMSD, 96 hours REM sleep deprivation period; Rec. Recovery period. The white back panels indicate the light phase and the shaded ones, the dark phase. *- different from baseline, effects of day are indicated by connecting lines above the symbols. ANOVA, followed by Newman-Keuls, *P* ≤ .05.

**Figure 3:**
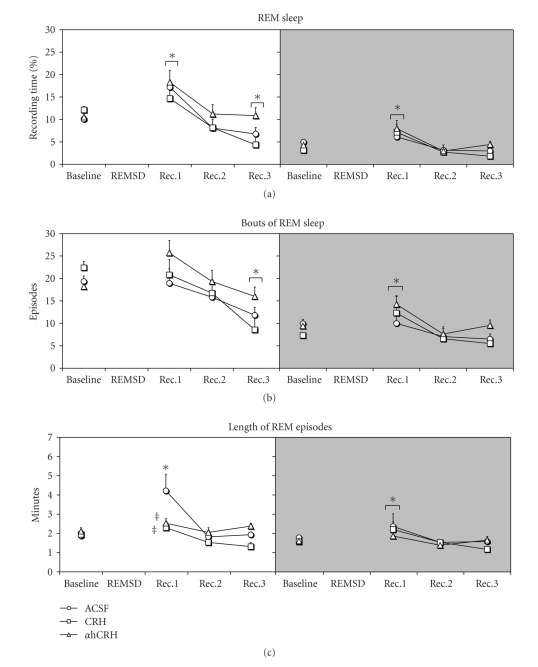
*REM Sleep*. Data were obtained in recording periods of approximately 11 hours during the light and dark phases. ACSF, artificial cerebrospinal fluid; CRH, corticotrophin-releasing hormone; *α*hCRH, alpha helical CRH_9−41_; REMSD, 96 hours REM sleep deprivation period; Rec. Recovery period. The white back panels indicate the light phase and the shaded ones, the dark phase. *- different from baseline, ^‡^- different from ACSF group. Main effects of day are indicated by connecting lines above the symbols. ANOVA, followed by Newman-Keuls, *P* ≤ .05.

**Figure 4:**
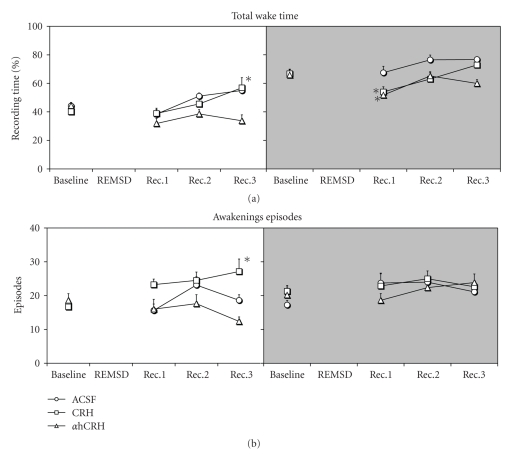
Awake. Data were obtained in recording periods of approximately 11 hours during the light and dark phases. ACSF, artificial cerebrospinal fluid; CRH, corticotrophin-releasing hormone; *α*hCRH, alpha helical CRH_9−41_; REMSD, 96 hours REM sleep deprivation period; Rec, Recovery period. The white back panels indicate the light phase and the shaded ones, the dark phase. *- different from baseline. Main effects of day are indicating by connecting lines above the symbols. ANOVA/Newman-Keuls, *P* ≤ .05.

**Figure 5:**
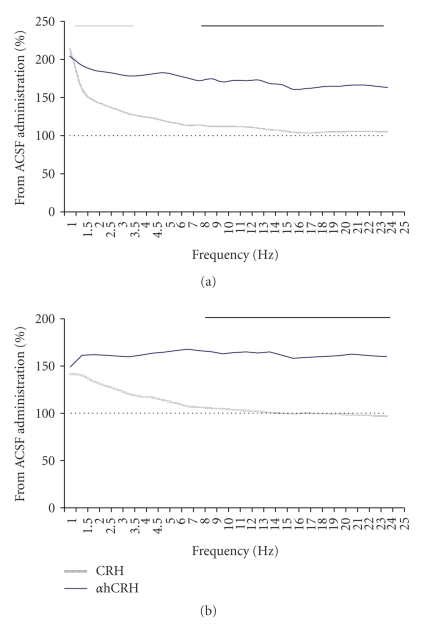
*Spectral Power During Total NREM on the 2nd (a) and 3rd (b) Recovery Dark Phases*. Results are expressed as percentage of ACSF-treated group, obtained from the mean power of each spectra band. ACSF, artificial cerebrospinal fluid; CRH, corticotrophin-releasing hormone; *α*hCRH, alpha helical CRH_9−41_. Grey line above graphics indicates differences of *α*hCRH from ACSF and black ones, the difference between the CRH and *α*hCRH treatments. Student's *t* tests, *P* ≤ .05.

**Figure 6:**
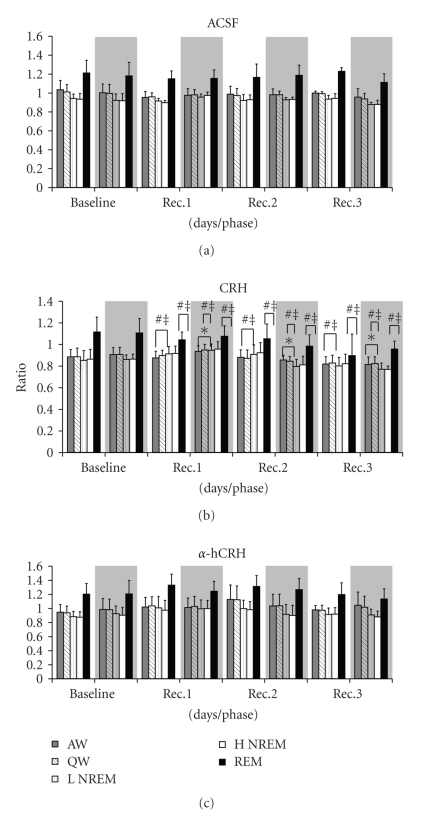
*Theta/Delta*. Theta/delta ratio is shown as mean of the total power in fast the *θ* (6.6–9.0 Hz) band divided by total power in fast *δ* (2.5–4.0 Hz) band, computed throughout ~11 hours period in the light and dark phases of the recovery period. ACSF, artificial cerebrospinal fluid; CRH, corticotrophin-releasing hormone; *α*hCRH, alpha helical CRH_9−41_; AW, active wake; QW, quiet wake; L NREM, low amplitude NREM sleep; H NREM, high amplitude NREM sleep; PS, REM sleep; Rec. Recovery period. The white panels indicate the light phase and the gray ones, the dark phase. *- different from baseline, ^#^-different from ACSF group, and ^‡^- different from *α*hCRH group. Main effects of sleep parameter are indicated by connecting lines above the bars. ANCOVA, followed by the Newman-Keuls test, *P* ≤ .05.
